# UAV-Based Estimation of Carbon Exports from Heterogeneous Soil Landscapes—A Case Study from the CarboZALF Experimental Area

**DOI:** 10.3390/s16020255

**Published:** 2016-02-19

**Authors:** Marc Wehrhan, Philipp Rauneker, Michael Sommer

**Affiliations:** 1 Leibniz Centre for Agricultural Landscape Research (ZALF), Institute of Soil Landscape Research, Eberswalder Straße 84, Müncheberg 15374, Germany; sommer@zalf.de; 2 Leibniz Centre for Agricultural Landscape Research (ZALF), Institute of Landscape Hydrology, Eberswalder Straße 84, Müncheberg 15374, Germany; philipp.rauneker@zalf.de; 3 University of Potsdam, Institute of Earth and Environmental Sciences, Karl-Liebknecht-Str. 24-25, Potsdam 14476, Germany

**Keywords:** UAV, multispectral, VI, agriculture, carbon export, soil landscape

## Abstract

The advantages of remote sensing using Unmanned Aerial Vehicles (UAVs) are a high spatial resolution of images, temporal flexibility and narrow-band spectral data from different wavelengths domains. This enables the detection of spatio-temporal dynamics of environmental variables, like plant-related carbon dynamics in agricultural landscapes. In this paper, we quantify spatial patterns of fresh phytomass and related carbon (C) export using imagery captured by a 12-band multispectral camera mounted on the fixed wing UAV Carolo P360. The study was performed in 2014 at the experimental area CarboZALF-D in NE Germany. From radiometrically corrected and calibrated images of lucerne (Medicago sativa), the performance of four commonly used vegetation indices (VIs) was tested using band combinations of six near-infrared bands. The highest correlation between ground-based measurements of fresh phytomass of lucerne and VIs was obtained for the Enhanced Vegetation Index (EVI) using near-infrared band b_899_. The resulting map was transformed into dry phytomass and finally upscaled to total C export by harvest. The observed spatial variability at field- and plot-scale could be attributed to small-scale soil heterogeneity in part.

## 1. Introduction

The application and enhancement of remote sensing methods and sensors have led to a better understanding of how leaf properties (age, shape, nutrient and water status) affect leaf reflectance and leaf emittance. Research on the contribution of plant canopy architecture, solar illumination conditions and soil reflectance to total canopy reflectance resulted in improved estimates of canopy parameters such as leaf area, standing phytomass, crop type and yield [1,2]. These techniques and methods can provide a valuable contribution to the analysis of processes controlling the carbon budget of agricultural landscapes.

Recently, Leaf Area Index (LAI) derived from multi-temporal broad-band optical and microwave remote sensors, has been assimilated successfully in carbon cycle modeling. LAI values of winter wheat were used to update the simulated LAI of a process-based model of cereal crop carbon budgets to improve daily net ecosystem exchange (NEE) fluxes and at-harvest cumulative NEE at the field-scale of different European study sites [3]. Time-series of Landsat TM and ETM+ data were used for leaf chlorophyll (Chl) retrieval to improve model simulations of gross primary productivity (GPP) of corn. The satellite-based Chl estimates were used for parameterizing the maximum rate of carboxylation (V_max_), which represents a key control on leaf photosynthesis within C_3_ and C_4_ photosynthesis models [4]. These approaches of data assimilation are promising but rely on the availability of remote sensing data. In the case of required time-series or time-sensitive applications such as management decision support, crop stress or erosion related events, most remote sensing platforms provide unfavorable revisit times (>5 days). In addition, the quality and finally the exploitability of satellite imagery depends on current weather conditions during image acquisition. On the local scale, the use of optical and thermal remote sensing sensors mounted on satellites and manned airborne platforms is either limited in spatial and spectral resolution or suffer from high operational costs [5].

The development of Unmanned Aerial Vehicles (UAVs) is an opportunity to overcome some of these limitations. The technical progress in the development of sensors, embedded computers, autopilot systems and platforms enables the construction of lightweight remote sensing systems with user defined flight intervals. For multitemporal data acquisition with high spatial resolution, these systems are much more economical than manned aircraft and allow a more flexible mission design than with the use of satellites. However, the challenge of using UAVs for environmental research is less their operational use but (i) the generation of radiometric and geometric corrected imagery; and (ii) the conversion of the spectral information to vegetation biophysical parameters. Previous studies focused on these two essential aspects for different sensors mounted on UAVs. Numerous procedures were applied to reduce the impacts of noise [6], radial illumination fall-off (vignetting) [6,7,8], lens distortions [5] and bidirectional reflectance effects [7] on imagery captured by different non-calibrated charge-coupled device (CCD) or complementary metal-oxide-semiconductor (CMOS) sensors. For the conversion of the pre-processed digital numbers (DN) into at-surface reflectance the above-mentioned authors applied an empirical line approach using a linear transformation derived from ground-based reflectance measurements of calibration targets and the respective image DNs.

Several UAV-based studies performed over agricultural fields were conducted with the aim to estimate vegetation biophysical parameters either by object-based image analysis [9] or by using vegetation indices (VIs) [7,10,11]. VIs are linear, orthogonal or ratio combinations of reflectance calculated from different wavelengths of the visible (VIS) and near-infrared (NIR) part of the electromagnetic spectrum [12] and widely used proxies for temporal and spatial variation in vegetation structure and biophysical parameters of agricultural crops [13]. Numerous VIs have been developed in the last decades to improve the relationships between the spectral response and the respective characteristics of vegetation canopies such as net primary production, LAI, vegetation fraction, chlorophyll density or the fraction of absorbed photosynthetic active radiation (FAPAR) [14,15,16]. Most VIs suffer from strong non-linearity and sensitivity to external factors such as solar and viewing geometry, soil background and atmospheric effects [17]. Apart from limitations caused by insufficient spectral resolution of available sensors, most improvements refer to the reduction of these external factors. Jiang *et al.* [18] give a brief review of different VIs, their limitations and proposed improvements.

Lelong *et al.* [7] implemented different VIs in generic relationships to successfully estimate LAI and nitrogen uptake of wheat varieties grown on micro-plots. Zarco-Tajeda *et al.* [10,11] demonstrated that narrow-band VIs derived from multi- and hyperspectral imagery captured during different UAV missions enable the detection of chlorophyll fluorescence emission variability as a stress status indicator of olive, peach and citrus trees. Although there is a growing demand on quantitative and spatially consistent data of major components of the carbon budget [19,20], to our knowledge, UAV-based remote sensing have not been used for the estimation of the C export by harvest of crops to date. Apparently easy to achieve, considerable uncertainties arise from the spatio-temporal variation of environmental factors (soils, weather conditions) and harvesting techniques (crop residues) [19].

In this case study we examined the potential of narrow-band multispectral imagery to estimate the C export of lucerne (Medicago sativa) from plots in different terrain positions and soil types. The output of a workflow for data pre-processing, a calibrated orthorectified image mosaic, was used to examine the predictive accuracy of the normalized difference vegetation index (NDVI) [21], the transformed soil-adjusted vegetation index (TSAVI) [22], the two-band vegetation index (TBVI) [23] and the enhanced vegetation index (EVI) [24] for fresh phytomass of lucerne. The total C export was then calculated from relationships between ground-based measurements of phytomass and carbon content of lucerne. Finally, yield data collected at each harvest date were used to estimate the yearly C export.

## 2. Methods

### 2.1. Study Area

**Figure 1:**
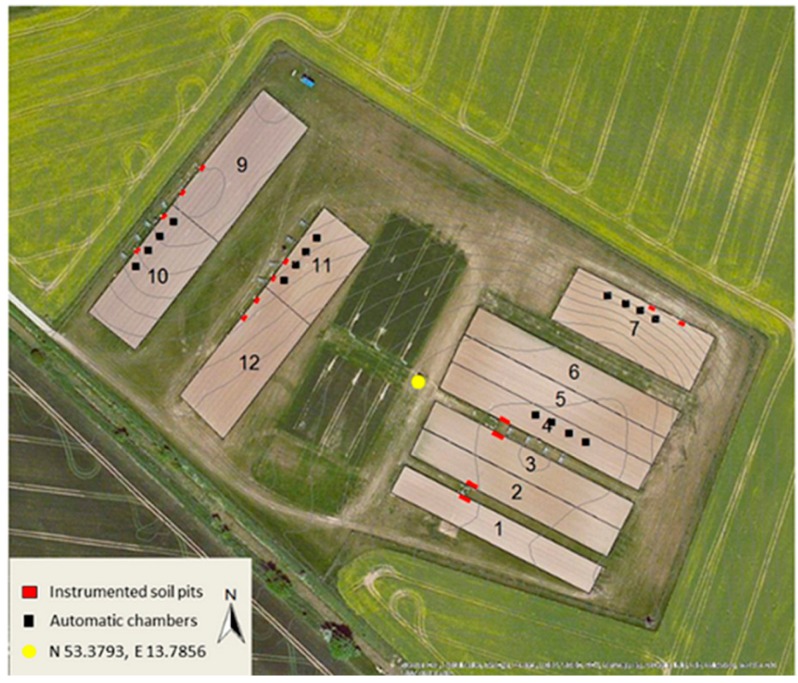
The CarboZALF experimental area near Dedelow (NE Germany): plot design and instrumentation.

UAV imagery was acquired in the Federal State of Brandenburg (NE-Germany) at the CarboZALF experimental area (53.3793N, 13.7856E) (Figure 1). The subcontinental climate is characterized by a mean annual air temperature of 8.7 °C and a mean annual precipitation of 483 mm (1992–2011, ZALF Research Station Dedelow). The 6 ha research area is embedded in a hummocky ground moraine landscape characterized by intense agricultural land use. Past and recent soil erosion by water and tillage leads to a very high spatial variability of soils and related growth conditions for crops. Only 20% of the region are unaffected by soil erosion. The 12 plots are extensively instrumented with the aim to conduct a long-term study (>10 years) on the impact of climate change, management and different soil types on gas exchange, carbon budget and carbon stocks of arable land in glacial landscapes [25]. The soils of the 6 ha experimental area represent a full gradient in erosion and deposition, namely a non-eroded Albic Cutanic Luvisols (plots 1–6), strongly eroded Calcic Cutanic Luvisols (plots 11–12), extremely eroded Calcaric Regosols (plot 7), and a colluvial soil, *i.e.*, Endogleyic Colluvic Regosols (Eutric) over peat (plots 9–10). In 2014, lucerne was grown on eight plots while corn and sorghum were grown on the remaining plots.

### 2.2. UAV-Platform Carolo P360

**Figure 2:**
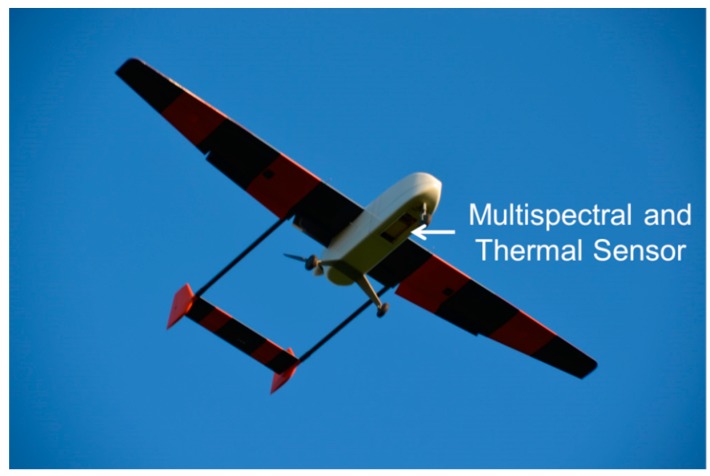
The Carolo P360 unmanned aerial vehicle (UAV) during a mission with open landing gear doors.

The Carolo P360 is a fixed wing construction, developed by the Institute of Aerospace Systems of the Technical University Braunschweig (Figure 2). With a wingspan of 3.6 m and a takeoff weight of almost 22.5 kg including the complete battery set, the UAV is capable of carrying an additional payload of approximately 2.5 kg. The battery set consists of two Lithium-Polymer (LiPo) batteries (each 10 cells and 10 Ah) for the electric drive motor, two LiPos (each 2 cells and 3.55 Ah) for the autopilot system including servo actuators and one LiPo (4 cells, 3.55 Ah) for the payload (sensors and control unit). The payload, mounted inside the fuselage, is protected during takeoff and landing by landing gear doors, opened and closed via radio control. Due to the size of the opening, the dimension of the sensor optics is limited to 10 cm width and 24 cm length. The battery set allows flight durations of approximately 40 min at ground speeds between 20 and 30 m·s^−1^ including the time for climbing and landing. However, for security reasons a recommended ground speed should not fall significantly below 24 m·s^−1^.

The combination of a strong electric motor (9.5 KW), large wheels and a rigid, spring mounted landing gear enables the UAV to takeoff and land on short airstrips (length: 70 m, width: 30 m including reserve) with rough surfaces. Takeoff, climbing and landing have to be performed manually by a pilot via radio control. During the autonomous flight, the task of the backup pilot is to observe and, in case of an emergency, abort the autonomous flight and land manually. The backup pilot is mandatory due to regulations of the national civil aviation authorities (CAA).

The autonomous flight of the UAV is controlled by a MINC autopilot. Navigation filter and algorithms utilize data from the inertial measurement unit (IMU) and GPS measurements. IMU measurements are combined with long time stable and precise but less frequent GPS measurements in a discrete error state Kalman filter. This algorithm requires the GPS signal of less than four satellites [26]. Extensions of the MINC system include a flight data recorder that stores collected IMU and GPS data on a 256 Mb MM card in a 2 s interval for further processing.

### 2.3. Mounted Sensors

Sensors mounted on UAVs are limited in their dimensions and weight. Despite the use of lightweight materials, a reduction in manufacturing quality, data storage capacity and on-board processing features is inevitable [6]. However, the development of miniaturized electronic components in the last decades enables scientist to mount a variety of sensors on UAVs for small-scale remote sensing applications. The Carolo P360 is equipped with two sensors, a multispectral and a thermal sensor. However, thermal sensor data were not used in this study.

**Figure 3:**
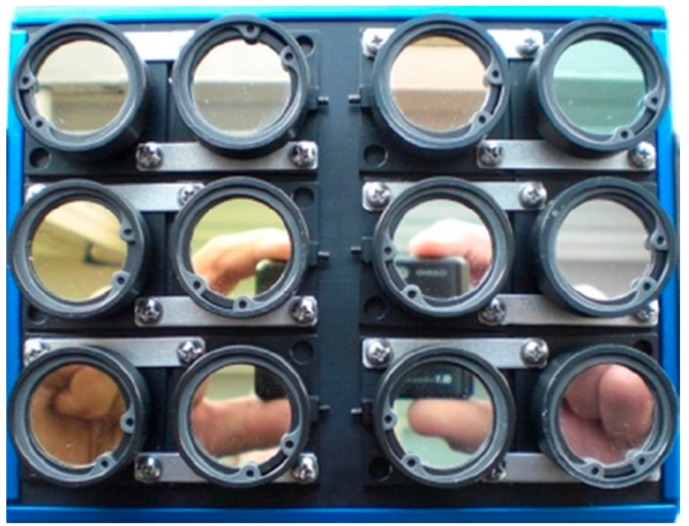
Tetracam Inc. miniature multi-camera array Mini-MCA 12 with mounted narrow-band (10–40 nm) filters that cover the spectral range between the visible and the near-infrared light (470–953 nm; both center wavelengths).

The core of the sensor equipment is a 12-band miniature multi-camera array Mini-MCA 12 (MCA hereafter) (Tetracam Inc., Chatsworth, CA, USA). The compact modular construction of the MCA integrates two basic modules into one rugged chassis. Each module consists of an array of six individual CMOS sensors (1280 × 1024 pixels; pixel size 5.2 µm), lenses (focal length 8.5 mm) and mountings for user definable band-pass filters (Figure 3). Images can be stored on 2 GB CF cards for every sensor either in DCM or RAW format. The PixelWrench2 software (Tetracam Inc., Chatsworth, CA, USA), shipped with the camera, enables the conversion of those images into single/multiband TIF or JPG format. Camera settings can be modified and enable the user to select camera exposure times between 1 ms and 20 ms (1 ms increment) and store images at a dynamic range of either 8 or 10 bit. In order to find an adequate exposure time, several ground-based tests prior to this study were performed at blue sky conditions over vegetation plots. A high dynamic range for most bands and no saturation at the same time was found for a fixed exposure time of 4 ms, which was then used in this study. The dynamic range was set to 10 bit.

The interchangeable band-pass filters, manufactured by Andover (Andover Corp., Salem, NH, USA), were selected prior to delivery, based on their center wavelengths and bandwidths. The 12 narrow-band filters cover, almost equally distributed, the spectral range from visible to near infrared wavelengths with focus on the characteristic reflectance features of healthy vegetation including the chlorophyll absorption band around 650 nm, the red-edge region between 680 nm and 730 nm and one of the water absorption bands around 950 nm. The bandwidth (full width at half maximum FWHM) varies from 9.1 nm to 40.8 nm with increasing bandwidths towards larger wavelengths. The filter configuration and characteristics are given in Table 1.

### 2.4. Ground Control Software

**Figure 4:**
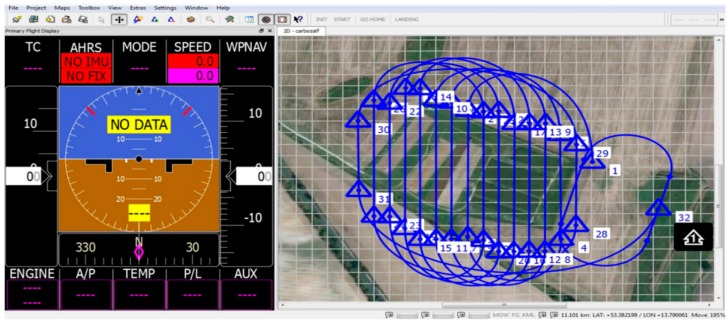
Screenshot of the MAVCDesk software. (**Left**) Primary flight display (not active) showing UAV status information; (**Right**) Visualization of the flight path across a map of the CarboZALF experimental area.

The ground control software MAVCDesk enables the operator to plan and control a mission. The planning of an autonomous flight encompasses the definition of waypoints, flight altitude, ground speed and a waypoint sequence mode. All required mission settings are finally transmitted to the autopilot via telemetry. During the mission, the telemetry antenna serves as a receiver for important UAV status information, which enables a visual position control (Figure 4). In the case of irregular behavior of any important flight parameter, the operator instructs the backup pilot to switch over to manual control.

### 2.5. Mission Settings

The UAV mission was conducted on 27 August 2014 under clear sky conditions and low wind speeds. The ground sampling distance (GSD) of 0.1 m was chosen as a compromise of flight altitude, cruising speed, shutter interval and high spatial resolution. The recommended cruising speed of the UAV is 25 m·s^−1^ and the camera shutter interval is fixed at a rate of 2 s. Sufficient overlap of consecutive images is necessary for post-processing of the imagery. Thus, we chose a flight altitude of 163 m, resulting in an overlap of approximately 50% in flight direction. To achieve a sufficient across flight overlap of at least 60%, we selected a distance of 40 m between the flight paths. Twenty-six waypoints were predefined, each marking a start- and endpoint of 13 parallel flight paths with a total length of 5.8 km excluding the loop lines.

### 2.6. Image Processing

The final goal of post-processing of recorded MCA imagery is the conversion of measured digital numbers (DN) into georeferenced at-surface reflectance images. This multistage procedure consists of three major components: (i) radiometric image correction; (ii) transformation of sensor coordinates into a geographic coordinate system and image alignment; and (iii) absolute radiometric calibration. The radiometric image correction includes noise reduction, correction of sensor-based illumination fall-off (vignetting) and lens distortion. The transformation of sensor coordinates includes the fusion of recorded GPS measurements with collected images, band-wise automated aerial triangulation (AAT), the minimizing of remaining geometric distortions and the alignment of the 12 single bands to one multispectral image using ground control points (GCPs). Radiometric calibration is the conversion of measured DNs into at-surface reflectance. For image correction we followed a practical approach proposed by Kelcey and Lucieer [6], developed for a single image captured by a Mini-MCA 6. They used dark offset imagery to create average noise corrections and images of a homogeneous illuminated near-Lambertian white surface to create a per-pixel flat-field correction factor. The procedure incorporates correction techniques proposed by Mansouri *et al.* [27].

#### 2.6.1. Noise Reduction

Noise is defined as unwanted electrical or electromagnetic energy that degrades the quality of signals and data. In the case of a CMOS-based camera, noise complies with all temporal and systematic errors added to a recorded signal during image acquisition. Noise is introduced both by the sensor (e.g., non-uniform pixel responses) and the electronics (e.g., electrical interferences) that amplify the output signal of the sensor for digitization [27]. Due to a random component, it is impossible to calculate the precise proportion of sensor noise to sensor signal within an image. Prominent examples are periodic noise, checkered patterns and horizontal band noise caused by the progressive shutter of CMOS sensors [6].

**Figure 5:**
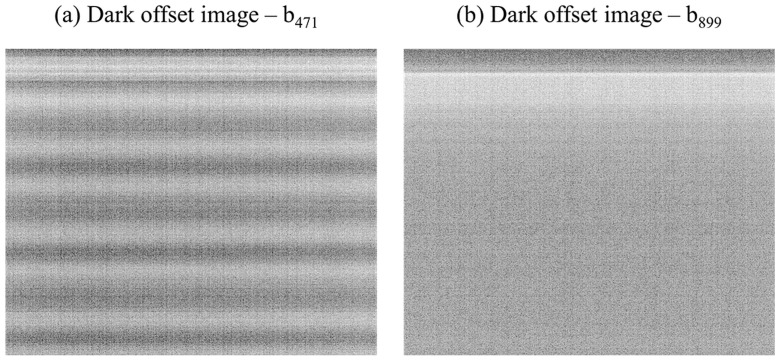
(**a**) Dark offset image of b_471_ showing periodic noise and progressive shutter band noise; (**b**) Dark offset image of b_899_ showing a global checkered pattern and progressive shutter band noise.

The dark offset subtraction technique is a statistical image based approach, which reduces the noise component of an image by subtracting a dark offset image. A dark offset image represents the average per-pixel noise and is generated by multiple repetitions in a completely darkened environment. The dark offset imagery was created for each sensor of the MCA in a darkened room with black painted walls. In addition, the MCA lenses were covered with black cardboard. For each of the 12 sensors, a total of 120 images at exposure level of 4 ms were taken to calculate the per-pixel average dark offset. The examples in Figure 5 show the dark offset images for bands 1 and 11 with strong periodic noise features in b_471_ (Figure 5a), a global checkered pattern in b_899_ (Figure 5b) and the overlapping horizontal progressive shutter band noise visible in both images.

#### 2.6.2. Vignetting Correction

The effect of radial fall-off of light intensity from the center towards the periphery in photographed images is known as vignetting. Different sources of vignetting contribute to a progressive reduction of irradiation across the image plane and may cumulate up to 60% at the periphery of an image. Although lens manufacturers go the limit to what is technically feasible, the geometry of the sensor optics contributes most to this effect [6,28]. In order to minimize the vignetting, an image based correction method was applied to each of the 12 bands of every single image captured during the mission. The method basically uses a look-up table (LUT) for each band, composed of correction factors for each pixel derived from flat field imagery. The proper generation of flat field imagery requires an evenly illuminated white surface with Lambertian properties and constant spectral characteristics. However, for practical considerations, a white surface with near Lambertian properties may serve to generate acceptable flat field imagery.

Numerous artificial white and black materials (various types of paper, rubber and plastic) were examined regarding their spectral properties. A halogen lamp was used for an even illumination of the targets and an ASD FieldSpec 4 Wide-Resolution spectrometer (ASD Inc., Boulder, CO, USA) was used to collect spectral reflectance data. The instrument measures the spectral radiance (W·m^−2^·sr^−1^·nm^−1^) over the wavelength range of 350–2500 nm with a spectral resolution of 3 nm at 700 nm and 30 nm at 1400 nm and 2100 nm, respectively. In order to yield the relative reflectance of a target (vegetation, calibration panels, soil), the measurement of the spectral radiance from a reference panel with Lambertian characteristic and constant spectral properties over VIS and NIR wavelengths was required. Therefore, a Spectralon^®^ reference panel was mounted on a tripod for collecting reference spectra prior to every single measurement of a target. Without fore optics, the bare fiber has a 25° field of view.

A matt white Bristol Cardboard with a density of 625 g·m^−2^ was found to show the highest and most uniform reflectance over the same wavelength range from 466 nm to 978 nm that is covered by the filter configuration of the MCA.

Vignetting imagery was created under diffuse illumination conditions using the matt white Bristol Cardboard. The camera was operated manually at a distance of approximately 1 m with the sensors pointing downwards to the Cardboard. Between each triggering the orientation of the camera has been changed slightly to minimize possible heterogeneities on the surface of the cardboard. During image acquisition we ensured that the cardboard completely covered the field of view of all 12 bands. In a first step, the per-pixel average was calculated from a total of 10 images for each of the 12 sensors at different exposure levels, followed by a subtraction of the respective dark offset imagery.

**Figure 6:**
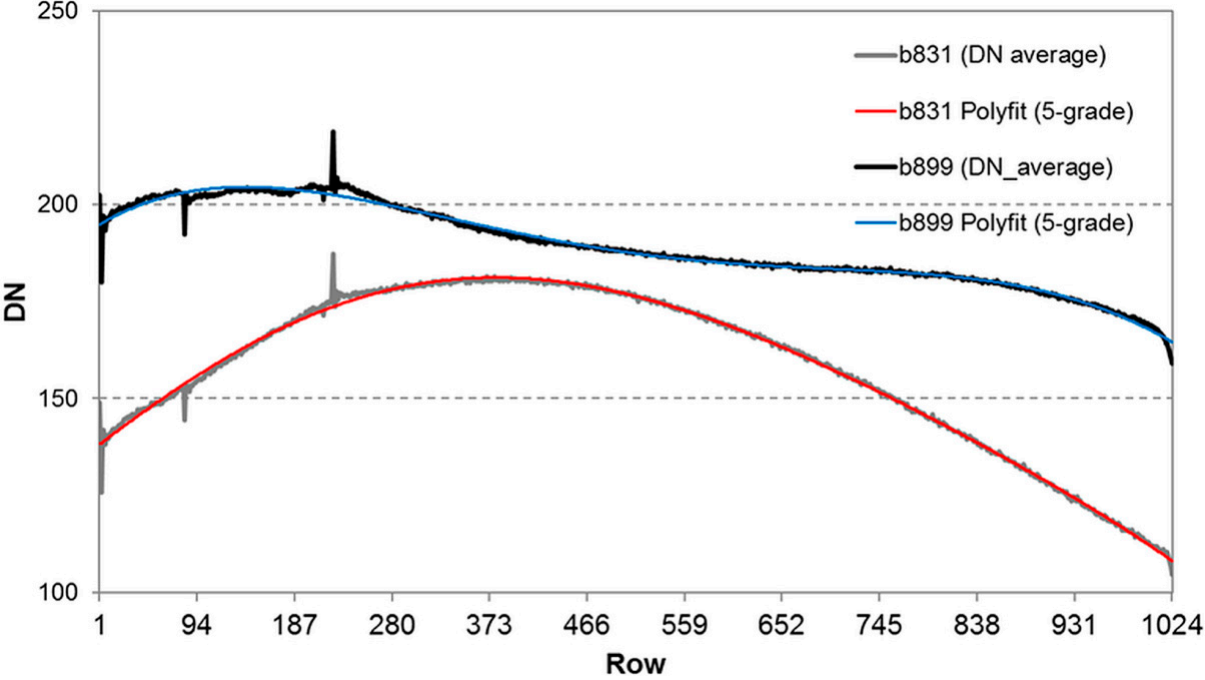
Row-wise average and 5-grade approximation of band-noise affected flat-field images of b_891_ and b_899_.

To account for the horizontal band noise induced by the progressive shutter of the camera, a shutter correction factor has been calculated in a second step. Each flat field image has been averaged along the y-axis (row-wise). While most profiles showed a behavior that could be approximated by a 3-grade polynomial function, satisfying approximation for b_761_, b_899_ and b_953_ could only be achieved by a 5-grade polynomial function. For the sake of consistency, we used a 5-grade polynomial for the approximation in all 12 bands. Figure 6 shows the y-axis average and its approximation for two examples, b_831_ and b_899_, respectively. For each row, the shutter correction factor has then been calculated by dividing the row-wise average by the approximation. Assuming a multiplicative row-wise brightness modification of the shutter, each row of the average flat field image was then multiplied by the respective correction factor.

**Figure 7:**
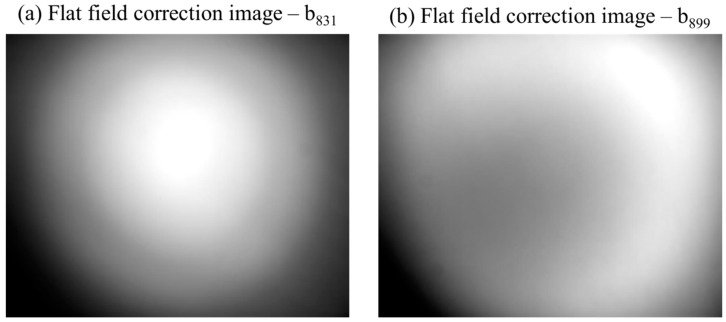
(**a**) Flat field image generated for vignetting correction of b_831_; and (**b**) for vignetting correction of b_899_.

Finally, the correction factor LUT for each sensor was then calculated by dividing all pixel values of the flat field imagery by the maximum pixel value that occurred in the respective image assuming the maximum value (the brightest pixel) to be an unaffected representation of the measured radiance. Figure 7 depicts an example for flat field images generated for b_831_ and b_899_.

**Figure 8:**
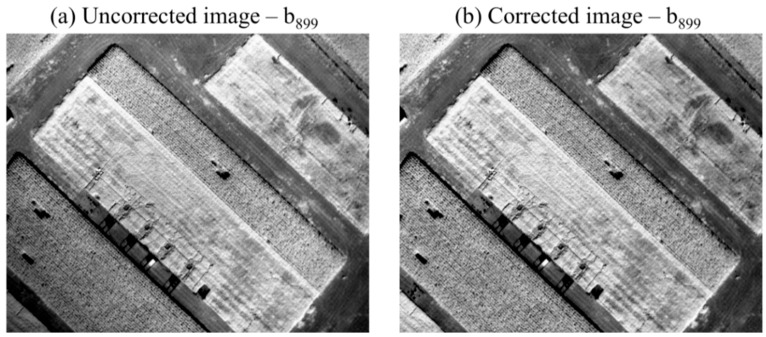
(**a**) Example for an uncorrected image (RAW format) recorded in b_831_; and (**b**) the respective image after noise reduction and consecutive vignetting correction.

The example in Figure 8 demonstrates the effect of the consecutive correction steps (noise and vignetting) to an uncorrected single image recorded in b_831_. Due to its relatively small contribution, noise reduction is almost invisible in the resulting image. However, the successful correction of the vignetting effect, especially in the upper and lower left corners, is evident.

#### 2.6.3. Lens Distortion Correction

Lens distortions arise from the symmetry of a photographic lens. The most frequent distortions are radially symmetric and known as barrel or pincushion distortion. In the case of a barrel distortion, image magnification decreases with distance from the optical axis. It increases in the case of a pincushion distortion. Both effects result in a radial displacement of measured per-pixel radiance.

A commonly applied correction technique for both types of distortion is the plumb-line approach described in the Brown–Conrady model [29], which is implemented in the PhotoScan-Pro V.1.2. software (Agisoft LLC, St. Petersburg, Russia). Since no correction factors are provided in the Exif tags of the imagery, the required internal and external orientation of each camera (band) is estimated automatically from the geometry of an image sequence during the image alignment process [30]. This first step in the PhotoScan-Pro workflow only requires the input of the focal length (8.5 mm) and the pixel size (5.2 µm) together with the GPS coordinates recorded for each individual image.

#### 2.6.4. Mosaicking and Georeferencing

**Figure 9:**
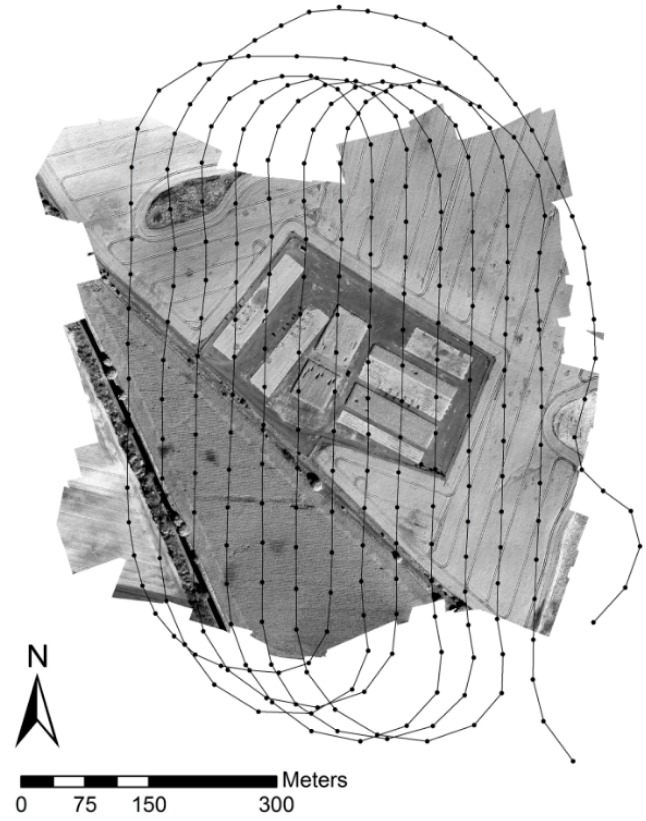
Image mosaic of b_761_. Overlay: Reconstructed flight path from recorded GPS locations (black dots).

Due to the proprietary nature of the software, the underlying algorithms are not known in detail. Anyway, the program workflow involves common photogrammetric procedures in a Structure from Motion (SfM) workflow, including the search for conjugate points by feature detection algorithms used in the bundle adjustment procedure, approximation of camera positions and orientation, geometric image correction, point cloud and mesh creation, automatic georeferencing and finally the creation of an orthorectified mosaic [31]. This workflow (lens distortion correction included) was applied to each of the 12 bands independently. The result of the workflow applied to b_761_ and the reconstructed flight path from the recorded GPS locations used for image alignment is illustrated in Figure 9.

The ERDAS Imagine software (Hexagon Geospatial, Norcross, GA, U.S.) was then used to improve the spatial accuracy and to transform the single bands to the local coordinate system ETRS 89 UTM 33 using precisely measured GCPs. Finally, the 12 bands were stacked to a single multispectral image.

#### 2.6.5. Radiometric Calibration

The retrieval of biophysical parameters of vegetation canopies requires an absolute calibration of the collected imagery because a recorded DN is not only a function of the spectral characteristics of vegetation or soils but also of environmental condition [32]. These include in particular the atmospheric conditions during the flight and the respective illumination geometry (solar zenith and sensor viewing angles). Several approaches exist to calculate the at-surface reflectance either by using radiative transfer models (RTM) or a combination of RTM and ground-based *in situ* measurements of the reflectance of a calibration target, a so-called in-flight calibration [33]. Both approaches require a well-calibrated sensor and on-site measurements of the atmospheric conditions at the date of image acquisition. To overcome these requirements, attempts were made to establish linear relationships between ground-based reflectance measurements and recorded DNs [34]. This empirical line approach accounts for both the influence of illumination geometry and atmosphere [32]. Prerequisite is the availability of low and high reflectance targets, with homogenous spectral characteristics over the wavelength range covered by the sensor. The approach has been applied successfully to different former UAV missions using the MCA [5,6,8] and is well suited for UAV remote sensing applications for several reasons: (i) the spectra of different materials potentially suited as targets can be examined prior to UAV missions; (ii) the spectra of selected calibration targets can be measured close to the time of image acquisition; and (iii) due to the high spatial resolution of images, the dimensions of the targets remain small and easy to carry.

**Figure 10:**
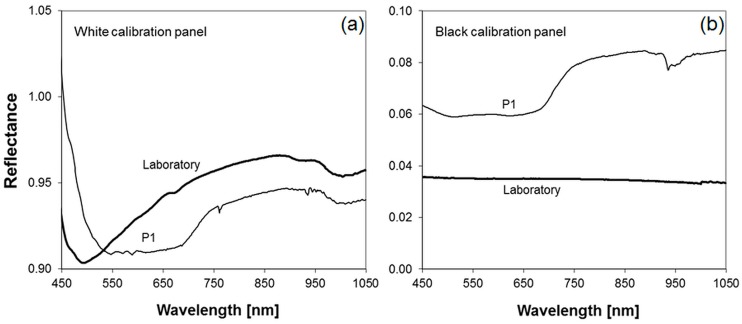
(**a**) Reflectance of the white calibration panel (matt white Bristol cardboard) from laboratory and field measurements at P1; (**b**) Reflectance of the black calibration panel (black cardboard) from laboratory and field measurements at P1.

The targets, in the following referred to as calibration panels, were constructed from thin chipboards with a dimension of 0.5 m × 0.5 m. The chipboards were coated with the matt white Bristol cardboard already used for the vignetting correction and with black cardboard with almost uniform low reflectance in the respective wavelength range. Five pairs of black and white calibration panels were placed in the four corners and the center of the study area. The spectral characteristics of each panel were measured during a time period of one hour around image acquisition. The measured reflectance of the white and black calibration panels tends to be lower under laboratory conditions than under clear sky conditions. This may be caused by non-Lambertian reflectance characteristics of the used materials which may also be a reason for the disparate reflectance values of the five black and white calibration panels over the relevant wavelength range (450–1000 nm). Slight angular deviations from the horizontal plane of the calibration panels showed undesired impacts on the reflectance especially of the white ones, partially exhibiting reflectance greater than one. Instead of using an averaged reflectance of all calibration panels, the reflectance of the one pair (P1), which comes closest to the respective laboratory reflectance was used (Figure 10).

The DN of the pixel with the highest value within the white calibration panels and the lowest value within the black calibration panels was plotted against the corresponding ground-based reflectance, calculated by averaging over the rounded FWHM bandwidths, for each of the 12 bands. The resulting 12 empirical lines were finally used to perform a band-by-band conversion of per-pixel DN into per-pixel reflectance.

### 2.7. Ground-Based Measurements

#### 2.7.1. Fresh, Dry Phytomass and Total Carbon Content of Lucerne

Lucerne belongs to the legume family and is usually grown for fodder production. Within crop rotations, lucerne is frequently grown for soil improvement due to its nitrogen-fixing properties. It was grown on eight equally managed plots, each fertilized with 300 kg·ha^−1^ phosphate and 110 kg·ha^−1^ potash. The date of the UAV mission corresponded with the growth stage of beginning flowering (BBCH—Code 61), and was conducted one day before the fourth harvest.

Fresh phytomass was sampled at 22 permanent observation sites after collecting spectral data. Plants were cut at ground level within an area of 0.25 m^2^ from two locations close to the permanent sites. After weighing, the samples were chaffed in order to guarantee uniform drying. Dry phytomass and the corresponding water content were determined after oven drying at 60 °C until constant weight (48 h). This intermediate step is required to estimate the amount of carbon content of each of the samples.

The carbon content of green lucerne was determined in an earlier study (2013, beginning flowering) at the CarboZALF experimental area by the ZALF Institute for Landscape Biogeochemistry (data not published). Samples of lucerne were analyzed in the ZALF central laboratory according to standard methods (spectral elementary analysis, DIN ISO 10694:1995). The total carbon content ranges between 41% and 46%. The mean of 43% (SD = 0.4, N = 100) is in good agreement with a reported mean of 45% used for most diverse crops in regional studies [35,36] and was finally multiplied with the amount of dry phytomass calculated for each pixel.

#### 2.7.2. Total Carbon Content of Lucerne per Vegetation Period

In order to estimate the total C export of the entire growing season, the averaged total carbon per plot calculated from MCA imagery were multiplied by factors derived from time-series of independently collected ground-based measurements of dry phytomass. These samples were routinely collected before each of the four harvest dates at least four representative locations within the individual plots. To determine dry phytomass, 1 m^2^ of plants was cut at ground level and oven dried at 60 °C (until constant weight (48 h). The factors were determined by dividing the sum of dry phytomass of all harvest dates by the dry phytomass collected at the fourth harvest date.

#### 2.7.3. Spectral Response of Vegetation and Bare Soil

Spectral reflectance measurements of vegetation, calibration targets and bare soils were collected using an ASD FieldSpec 4 Wide-Resolution spectrometer. Since the distance between fiber optics and canopy was approximately 0.8 m, the collected spectra represented an average reflectance from a circle of 0.36 m in diameter. For compensation of slight movements of the fiber optics introduced by the operator while collecting the spectra, a number of 10 repetitions were set to default.

The sampling of vegetation spectra was performed between 11:00 a.m. and 1:00 p.m. local time, ±1 h before and after image acquisition. Forty-four spectra were taken at 22 permanent observation sites representing the natural variability of site properties. Each is represented by two spectral measurements for compensation of slight variations of standing fresh phytomass in its surrounding. The mean spectra of the two repetitions were then averaged to a single spectral response curve that represents the characteristics of the fresh phytomass at the site. The spectral measurements of the ten calibration panels (five white and five black panels) were taken during the same period (11:00 a.m. and 1:00 p.m. local time) as the vegetation spectra. The panels were placed in pairs (a white and a black) close to the four corners and the center of the study area.

**Figure 11:**
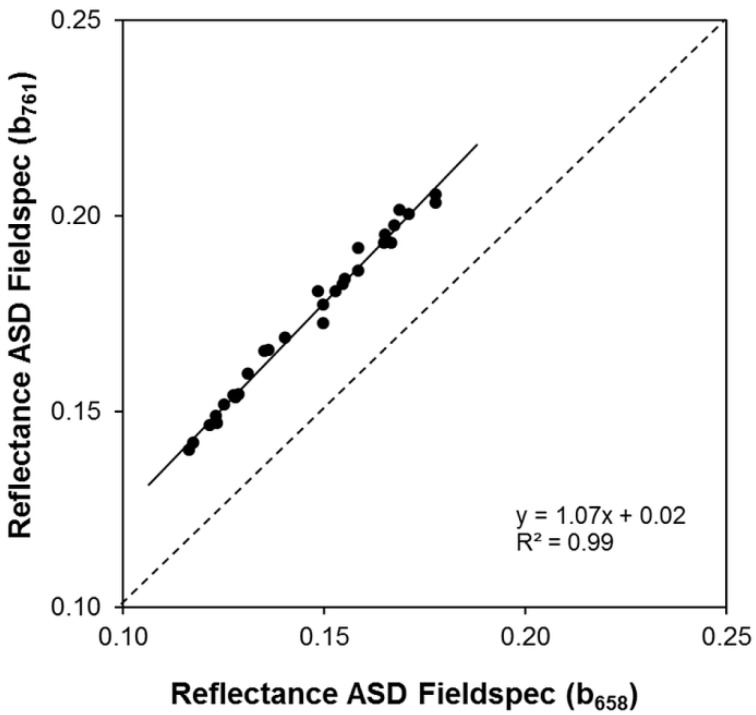
Relationship between ASD Fieldspec measurements of topsoil reflectance in the wavelengths corresponds to Mini-MCA 12 bands b_658_ and b_756_.

The spectral response of bare soil was collected at 18 September between 11:00 a.m. and 1:00 p.m. local time under clear sky conditions. Sample locations were identical to those selected for the collection of vegetation spectra. One day after the seeding of winter wheat, the topsoil showed a gentle surface roughness. From this, little influence on light scattering can be assumed. Topsoil conditions varied in moisture (7% to 12%) according to differences in terrain position and soil properties. The spectra were then used to calculate slope and intercept of the soil-line required for the calculation of the TSAVI. The best correlation (*R*^2^ = 0.99) between the b_658_ and one of the six NIR bands was found for b_756_ (Figure 11). The resulting parameters of the regression (*a* = 1.07; *b* = 0.02) were then used for the calculation of TSAVI.

### 2.8. Description and Calculation of VIs

The NDVI is an intrinsic vegetation index that simply accounts for the chlorophyll absorption feature in the red (R) and the structural information inherent in high NIR reflectance of a green vegetation canopy. It does not involve any external factor other than the measured spectral reflectance.

NDVI = (NIR − R)/(NIR + R)
(1)

The TSAVI incorporates slope and intercept of the soil line together with an adjusted coefficient to account for first-order soil background variation.

TSAVI = *a*(NIR − *a*R − *b*)/[(R + *a*(NIR − *b*) + 0.08(1 + *a*^2^)] (2)

where *a* is the slope and *b* is the intercept of the soil line. The value 0.08 is an adjusted coefficient.

The TBVI is a general formulation of the NDVI and was used to examine the predictive accuracy of other provided band combinations than used by the NDVI.

TBVI_i,j_ = (Ref_j_ − Ref_i_)/(Ref_j_ + Ref_i_) (3)

where i, j = 1, …., N, where N is the number of narrow bands and Ref is the reflectance measured in a narrow band.

EVI, originally developed as a standard satellite vegetation product for the Moderate Resolution Imaging Spectroradiometer (MODIS), combines atmospherically corrected blue (B), R and NIR reflectance with coefficients of an aerosol resistance term and a soil-adjustment factor. The use of the EVI has been motivated by studies reporting a trend to more linear relationships with vegetation biophysical parameters such as standing biomass and LAI of crops and a wider range of values at the same time [4,37].

EVI = 2.5 ((NIR − R)/(NIR + C_1_R − C_2_B + L)) (4)

where 2.5 is a gain factor. C_1_ and C_2_ are coefficients of the aerosol resistance term and L is the soil background reflectance adjustment where C_1_ = 0.06; C_2_ = 0.08 and L = 1.

In order to investigate the potential of the six available bands in the NIR, six variations of the NDVI, the TSAVI and the EVI were calculated. Variations of the TBVI were calculated for all band combinations except those covered by the NDVI variations (the R band in combination with the six NIR bands).

## 3. Results and Discussion

### 3.1. Radiometric Calibration

**Figure 12:**
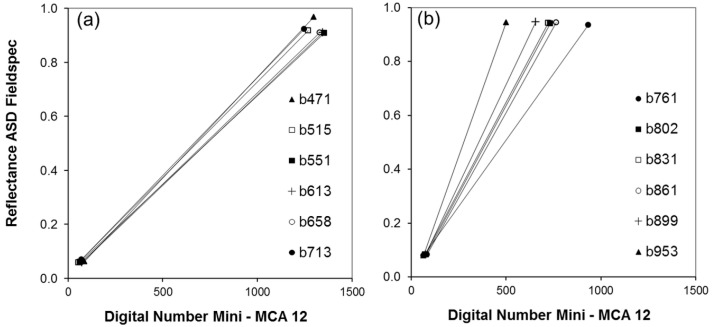
Relationship between ground measured reflectance of black and white calibration panels and the respective digital numbers acquired by Mini-MCA 12. (**a**) Bands 1–6; and (**b**) Bands 7–12.

The empirical line approach used for sensor calibration produces a set of 12 linear relationships between DNs and ground measured reflectance of a white and black calibration panel. Figure 12 shows the empirical lines for bands b_471_–b_713_ (Figure 12a) and bands b_761_–b_953_ (Figure 12b).

Raw imagery DNs of the white panels for bands 1–6 showed values close to saturation (DN 1024). After flat-field correction those pixels showed values higher than 1024. This is caused by flat-field correction factors >1, in cases where the white panel was situated close to the periphery of an image. Image DNs of the calibration panels and the corresponding ground measured reflectance are listed in Table 2 together with the respective regression.

**Figure 13:**
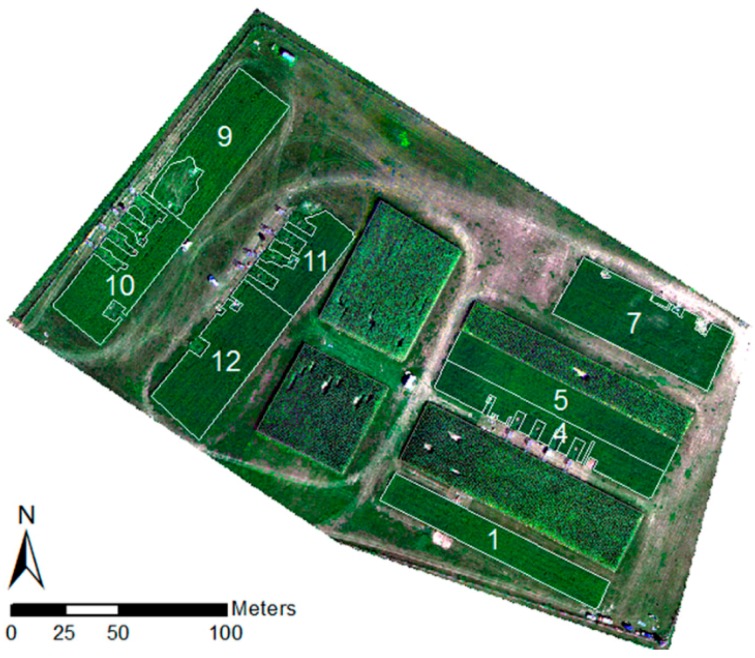
RGB composite image of the CarboZALF experimental area from calibrated Mini-MCA 12 bands b_658_, b_551_ and b_471_.

The spatial subset depicted in Figure 13 shows a composite from MCA b_658_ (R), b_551_ (G) and b_471_ (B) after calibration, band alignment, layer stacking and transformation from geographical coordinates (WGS 84) to projected coordinates (ETRS 89 UTM 33).

### 3.2. Empirical Line Quality Assessment

**Figure 14:**
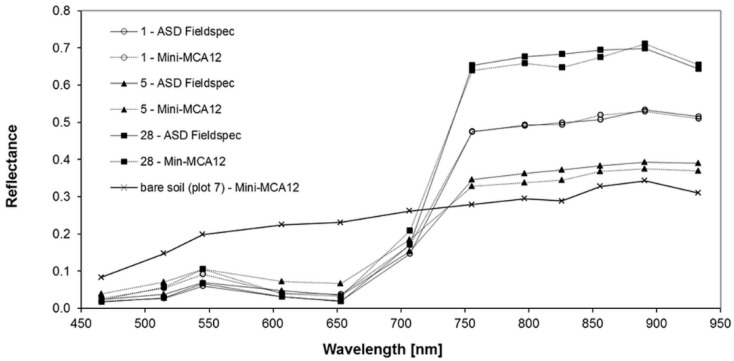
Comparison of the spectral response of lucerne extracted from calibrated Mini-MCA 12 bands with ground measured ASD Fieldspec reflectance and with bare soil reflectance (extracted from calibrated Mini-MCA 12 bands; ASD Fieldspec reflectance not available). The selected sites represent high (28), medium (1) and low (5) amounts of fresh phytomass of lucerne. The bare soil spectrum represents an area free of vegetation within plot 7.

An examination of calibrated MCA spectra and the corresponding ground-based measurement reveals good agreement. Generally, the spectra of both ground-based and calibrated MCA reflectance show the characteristic features of a green vegetation canopy with different amounts of biomass and soil covers (Figure 14). The depicted examples represent sites with high (28), medium (1) and low (5) amounts of fresh phytomass of lucerne.

Reflectance in the VIS is low in the blue and red domain and shows the characteristic peak in the green domain. After the transition from VIS to NIR wavelengths around 712 nm, NIR reflectance varies between 0.33 and 0.65 at 761 nm and between 0.38 and 0.71 at 899 nm. The water absorption band around 953 nm reveals differences between the spectra regarding the relative decline of the reflectance compared to b_899_. The example of a bare soil reflectance curve in Figure 14 indicates the general ability of calibrated MCA imagery to produce realistic spectra not exclusively for vegetation. The spectrum represents a small area free of vegetation within plot 7 and shows the typical monotonous increase of reflectance from VIS to NIR wavelengths within a realistic range of values.

However, the VIS spectral response of lucerne in calibrated MCA imagery is generally higher than the ground based reflectance. In the NIR domain, MCA acquired reflectance closely matches the ground-based measurements. Coefficients of determination reveal high correlations between ground-based and image reflectance for the six NIR-bands b_761_–b_953_, poor correlations for the four VIS bands b_471_–b_613_ and the NIR band b_713_ and a moderate correlation for the red band b_658_ (Table 3). The corresponding root mean square errors (RMSE) are low in terms of absolute reflectance but differ extremely in relation to the range of values in the respective bands. The mean relative error (MRE %) illustrate the discrepancy between the VIS (33%–104%) and the NIR bands (4%–23%). We assume the poor matching in the VIS bands are partially caused by the saturated pixels of the white panels used for the creation of the empirical lines. Additional greyscale panels in the medium reflectance range (0.3–0.7) would have been helpful to make the empirical line relationship more robust [38]. Nonetheless, additional sources of error might be present since other authors reported similar discrepancies for the VIS response of the MCA [39].

### 3.3. Ground-Based Measurements of Vegetation

**Figure 15:**
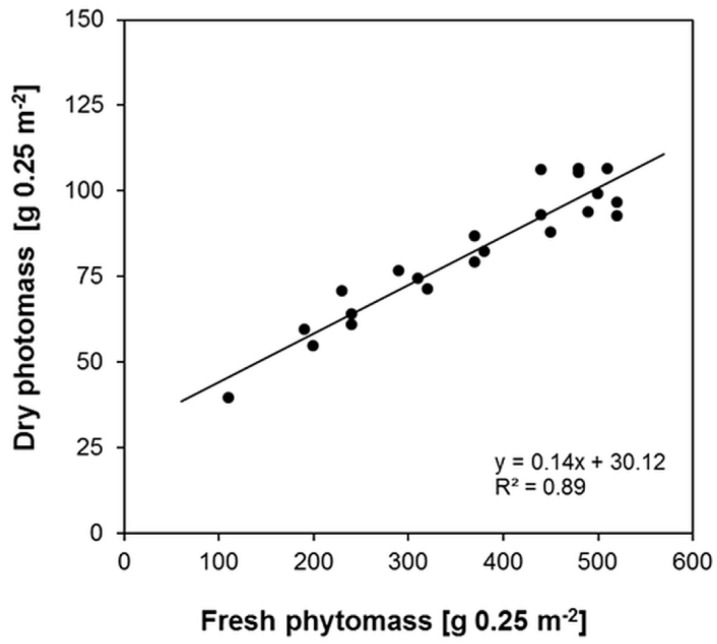
Relationship between fresh and dry phytomass of lucerne measured at the 22 permanent observation sites.

The different amounts of fresh phytomass of lucerne, sampled at 22 locations across the different plots reflect the spatial heterogeneity of naturally occurring site properties (terrain and soil). Although all plots were treated equally, the averaged fresh phytomass of each permanent observation site ranges between 440 g·m^−2^ and 2080 g·m^−2^. The overall mean is 1469 g·m^−2^ and the coefficient of variation (CV) is 34%. The amount of dry phytomass ranges between 158 g·m^−2^ and 426 g·m^−2^. The overall mean is 329 g·m^−2^ with a CV of 23%. The variation in the corresponding water content ranges between 282 g·m^−2^ and 1709 g·m^−2^. With a CV of 38%, the variation is similar to the variation in fresh phytomass. The statistic evaluation of the data shows a strong linear correlation (*R*^2^ = 0.89) between fresh and dry phytomass with a RMSE of 24 g·m^−2^ (Figure 15).

### 3.4. VI Performance

Coefficients of determination were calculated for the expected exponential relationships between ground measured fresh phytomass and all band combinations of the four VIs, as described above. The results indicate clear differences between the VIs and, with the exception of TBVI, only little differences between the variants using one of the six NIR bands for VI calculation. Regardless of the used NIR band, the best relationships are obtained for EVI, followed by TSAVI using the soil-line and NDVI. Coefficients of determination calculated for EVI range between 0.86 (b_761_ and b_953_) and 0.88 (b_802_, b_861_ and b_899_). Lower *R*^2^s can be observed for TSAVI and NDVI but again, MCA b_761_ and b_953_ (0.80 for TSAVI; 0.71 for NDVI) are less suited than b_802_ and b_899_ (0.82 for TSAVI; 0.72 for NDVI). Due to the construction of TBVI exclusively from NIR bands only five band combinations with b_953_ are possible. Low correlations were obtained when using combinations with b_761_ and b_861_ (*R*^2^ = 0.42 and 0.38, respectively), moderate correlations for the combinations with b_802_ and b_831_ (*R*^2^ = 0.65) and a high correlation (*R*^2^ = 0.77) for the combination with b_899_ (TBVI_b899/b953_ hereafter).

The small differences between the results obtained for EVI, TSAVI and NDVI variants can be explained by the low variation of canopy reflectance in the different NIR bands and the corresponding low reflectance in the R band (and B band in the case of EVI). The high correlation of the TBVI_b899/b953_ may be explained by the relatively large difference in reflectance compared to other NIR band combinations. The distance increases with higher amounts of fresh phytomass due to the maximum of NIR reflectance in b_899_ observed for all measured spectra and the relatively strong decline of the reflectance in b_953_, caused by the respectively higher absolute water contents. However, the highest correlations between the examined VIs and ground-based measurements of fresh phytomass were found for the variants using NIR band b_899_. Regardless the mathematical construction of VIs from available broad or narrow band sensors the disadvantage concerning the non-linearity caused by saturation effects, especially in the case of dense vegetation canopies, is still present. Saturation levels are reported in numerous studies using field measurements of biophysical canopy parameters or leaf and canopy radiative transfer model [17,40,41]. The studies compared the predictive power and stability of several narrow- and broad-band VIs for estimation of LAI under different environmental conditions (canopy architecture, soil background and illumination geometry). Values for NDVI of 0.90 and 0.75 for TSAVI when using a single soil line are typical for dense vegetation canopies (LAI > 2). The observed saturation effect for the relationship between VIs and fresh phytomass in this study is caused by the strong linear relationship between LAI and fresh phytomass (*R*^2^ = 0.88; LAI was measured simultaneously to fresh phytomass).

**Figure 16:**
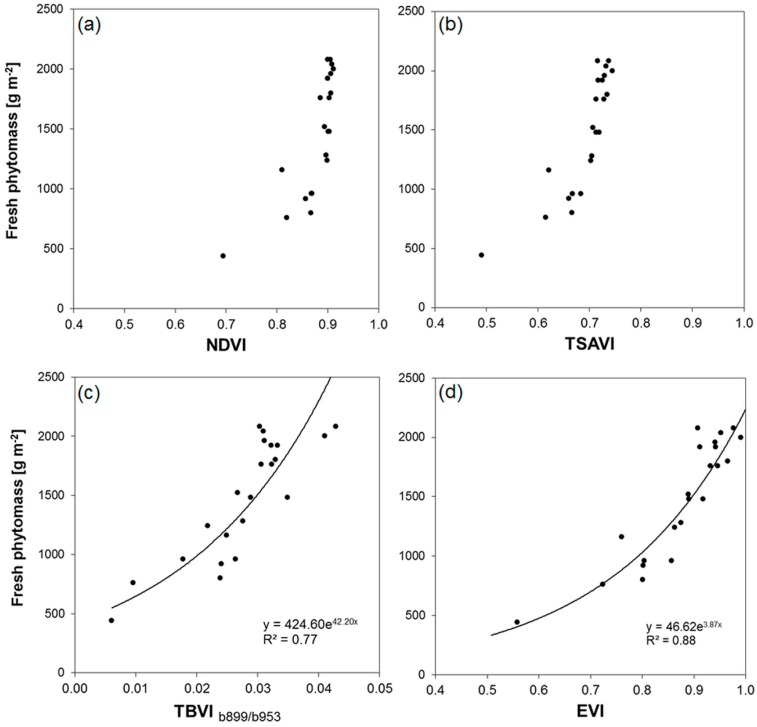
Relationships obtained between (**a**) NDVI; (**b**) TSAVI; (**c**) TBVI_b899/b953_; and (**d**) EVI constructed from VIS bands in combination with NIR band b_899_ (except TBVI_b899/b953_) and fresh phytomass of lucerne at the 22 permanent observation sites.

In this study, most NDVI values range in a narrow span between 0.89 and 0.91 when fresh phytomass exceeds 1200 g·m^−2^, which holds true for 15 out of 22 samples (Figure 16a). The same effect can be observed for the TSAVI where the same 15 samples range in a span between 0.70 and 0.74 (Figure 16b). These findings indicate that both NDVI and TSAVI are not reliable estimators for fresh phytomass of a dense green vegetation canopy typical for lucerne. In terms of *R*^2^ s, the TBVI_b899/b953_ performs better than the NDVI but worse than the TSAVI. The lower *R*^2^ compared with TSAVI is caused by a larger scatter in the data. This is probably the result of the small differences in the reflectance in b_899_ and b_953_. Consequently, results are more sensitive to remaining noise in the data after sensor calibration than the results for VIs calculated from NIR and VIS bands with large differences in reflectance. Nonetheless, TBVI_b899/b953_ exhibits a trend to more linearity than both NDVI and TSAVI (Figure 16c). Although developed for broad MODIS bands with the respective optimized coefficients to reduce impacts of soil and aerosol, EVI is the best predictor for fresh phytomass (Figure 16d). As reported in literature [4,37,42] the relationship is more linear and the range of values is wider (0.43) than observed for NDVI (0.25) and TSAVI (0.22). The RMSE and MRE for fresh phytomass using EVI are 193 g·m^−2^ and 11%, respectively.

### 3.5. Spatial Variability of Fresh Phytomass

**Figure 17:**
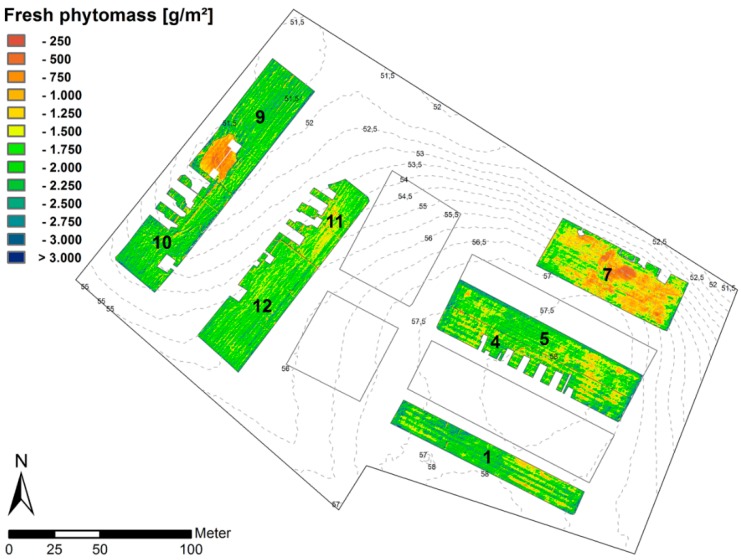
Spatial distribution of fresh phytomass of lucerne within the eight plots of the CarboZALF experimental area. Outclipped areas are disturbed areas due to experimental devices (autochambers, pathways, *etc.*).

The relationship between ground-based measurements of fresh phytomass and EVI calculated from calibrated MCA imagery was used in a first step to produce a map of fresh phytomass of lucerne for the eight plots of the CarboZALF experimental area (Figure 17). The high resolution enables a clear spatial differentiation of areas with extremely low (<250 g·m^−2^) and high amounts of fresh phytomass (>3000 g·m^−2^). The spatial patterns across and within the individual plots will be discussed in the context of total C export by harvest. The eye-catching area in the southwest corner of plot 9 with extremely low amounts of fresh phytomass relates to an inundated spot (two months) as a result of high rainfall in spring. This part will be excluded in the further evaluation.

### 3.6. Total C Export by Harvest—Quantities and Spatial Variability

**Figure 18:**
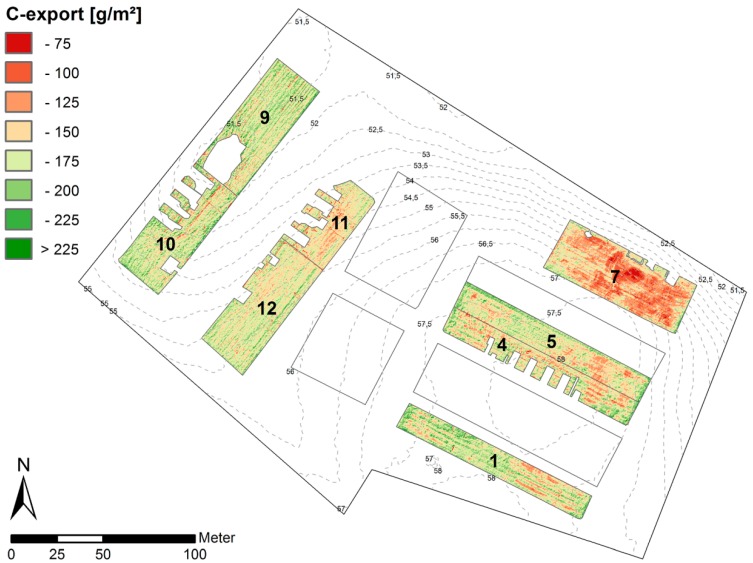
Spatial distribution of total exported carbon by harvest within the eight plots of lucerne at the CarboZALF experimental area.

To convert fresh phytomass into a map of total C export, the linear relationship between fresh and dry phytomass depicted in Figure 14 was used. The result was then multiplied by the averaged total carbon content (44%) to quantify the C export. Due to the linear transformation, the spatial patterns across and within the individual plots remain the same (Figure 18). The majority of values (99%) range between 75 g·m^−2^ and 225 g·m^−2^. In order to evaluate effects of terrain and prevalent soil type on the total C export, the median (M) for each individual plot was calculated (observations were normally distributed). The lowest C export is at plot 7 (M = 124 g·m^−2^), which represents an extremely eroded soil at steep slope. The C export from colluvial soils in the hollow (plot 9, M = 164 g·m^−2^; plot 10, M = 163 g·m^−2^) is only slightly higher than from non-eroded soils at the flat hilltop (plot 1, M = 162 g·m^−2^; plot 5, M = 154 g·m^−2^ and plot 4, M = 151 g·m^−2^). The internal spatial heterogeneity is higher at flat hilltop positions (plots 1, 4 and 5) and at steep slopes (plot 7). The plots in other terrain positions (plots 9, 10 11 and 12) appear more homogenous with coincidently higher phytomasses.

Although plots 12 and 11 are located in a similar flat slope position, the difference between the respective exports (M = 157 g·m^−2^ and 146 g·m^−2^ respectively) is higher than from other adjacent plots (9, 10 and 4, 5). This effect may be the result of a manipulation experiment that was conducted in 2010 to simulate landscape-scale erosion processes [25]. However, the interpretation of this effect is beyond the scope of this paper.

Weather conditions in 2014 were ideal for growing since plant growth was not limited by rainfall input. Therefore, differences across the plots are not very distinct. Generally, the observations match the spatial arrangement of soil types in the respective terrain positions, which is characteristic for this hummocky soil landscape. The Calcaric Regosol (plot 7) represents widespread extremely eroded soils with very dense parent material (glacial till) at 30 cm depth. Therefore, the rooting space is rather limited. The Endogleyic Colluvic Regosol (plots 9 and 10) in the hollow has the highest organic matter and nutrient stocks compared to other soils and shows local groundwater level is approximately 80 cm, hence additional water supply for an enhanced plant growth by capillary rise. The strongly eroded Calcic Cutanic Luvisol (plots 11 and 12) at midslope and the non-eroded Albic Cutanic Luvisols at the flat hilltop are generally fertile and characterized by high available water capacities and good root penetration.

### 3.7. Total C Export by Harvest Per Year—Temporal Trends and Spatial Variability

**Figure 19:**
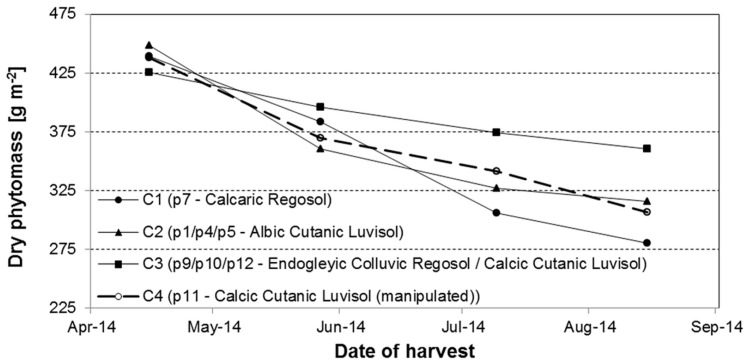
Temporal decline of above ground dry phytomass of lucerne between the first and fourth harvest in 2014.

For the estimation of the C export over the entire growing season, the samples collected at the four harvest dates were used (from monitoring program in 2014). While the relationship between terrain position/prevalent soil type and exported carbon by the fourth harvest shows a weak trend, the dependency becomes more obvious when the development of dry phytomass over the entire growing season is taken into account. The amounts generally decrease continuously from the first to the fourth harvest date. However, the differences between soils become obvious over time (Figure 19). The strongest decline can be observed for the extremely eroded soil (plot 7) hereafter referred to as C1. Whereas soils with additional water supply, either by groundwater (plots 9 and 10) or lateral water fluxes in 1.5m depth (plot 12, without manipulation), showed the lowest decline over time (C3). The non-eroded soils at the plateau (plots 1, 4, 5) and the manipulated plot 11 behaved intermediate (C2).

The averaged export of total carbon for the fourth harvest (27 August 2014) ranges between 124 g·m^−2^ from C1 and 161 g·m^−2^ from C3 (Table 4). With 156 g·m^−2^, C2 cannot be distinguished clearly from C3. Multiplying these values with the case specific factors calculated from the summed phytomass divided by the phytomass from the fourth harvest, the total exported carbon per year ranges between 624 g·m^−2^ from C1 and 718 g·m^−2^ from C2, which is slightly more than the 697 g·m^−2^ from C3. This is caused by the higher mean dry phytomass estimated from UAV imagery for the plots belonging to C2 (363 g·m^−2^ *vs.* 316 g·m^−2^).

Altogether, the differences are relatively small between the groups due to weather conditions in 2014, which were almost optimal for plant growth. Nevertheless, differences in site properties (terrain and soil) are known to result in quite different growth conditions [43,44] and seasonal and intra-annual changes in weather conditions affect the within-field variability of phytomass production [45,46]. Taylor *et al.* [47] reported higher within-field variation of crop yield in dryer years, which was spatially associated with soil properties. The effect was less pronounced in wetter years with adequate water supply, which was most recently confirmed in a study by Stadler *et al.* [48]. In addition, soil related within-field variability was found to be more pronounced and visible (beginning senescence) at the end of the growing season [44,45]. As a consequence, mapping of small-scale variability of crop characteristics and finally C-export is most effective in a narrow time window. UAV-based remote sensing meets all requirements for this purpose and helps to reduce time consuming and expensive ground-based measurement campaigns.

## 4. Conclusions and Outlook

In this case study, we presented a successful approach of how to use the combination of UAV-based high resolution remote sensing data and ground truth measurements for the estimation of the total C export by harvest of lucerne. The image pre-processing of the 12-band multispectral images has led to a considerable reduction of noise and vignetting effects. Together with the subsequent mosaicking and band alignment, the workflow is operational and will reduce the pre-processing time in future UAV missions. The conversion of recorded digital numbers to at-surface reflectance was only successful for the six NIR bands (b_761_-b_953_) and the R band b_658_. However, these were the most important bands for the calculation of the VIs used in this study. Nevertheless, improvements of the experimental design to obtain the empirical lines for absolute radiometric calibration of the remaining bands in the VIS are necessary.

The strong correlation between fresh phytomass of lucerne and the EVI (*R*^2^ = 0.88) using the NIR band b_899_ demonstrated the power of this VI even in the case of a dense green vegetation canopy. The map of the total C export revealed the potential of high spatial image resolution to: (i) map high small-scale variability within and across the different plots (75 g·m^−2^ and 225 g·m^−2^); and (ii) identify the spatial pattern as a result of different terrain positions and ascociated soil types.

Future UAV missions should include important annual crops such as winter wheat or corn. The temporal flexibility of the UAV should be exploited for intra- and inter-annual studies of the temporal carbon dynamics, coupled with research campaigns focus on other components of the carbon budget (e.g., gas exchange measurements). The fixed-wing Carolo P360 proved to be a valuable instrument for mapping vegetation parameters over small experimental areas. The high cruising speed and the potential flight endurance of at least 30 min. have the potential to map larger areas which cover the full spectrum of terrain position/soil type combinations in this heterogeneous soil landscape under equal imaging conditions.

Finally, the respective results should be coupled with other available sources of spatially consistent proximal and remote sensing data and assimilated in state of the art models to gain improved insight into the processes controlling the carbon budget of agricultural landscapes.

## Figures and Tables

**Table 1:** Filter configuration of the Mini-MCA 12 and optical properties of the mounted filters. For band 2 (b), no fact sheet has been provided (N/A).

Band	Center Wavelength (nm)	FWHM * Coordinates (Bandwidth) (nm)	Bandwidth (10%) (nm)	Peak Transmission (%)
b_471_	471	466.0–475.1 (9.1)	12.8	68.3
b_515_	515	N/A (≈10.0)	N/A	N/A
b_551_	551	545.5–555.6 (10.1)	14.8	56.4
b_613_	613	607.7–617.8 (10.2)	14.2	67.6
b_658_	658	653.4–662.9 (9.5)	13.6	69.2
b_713_	713	708.1–717.7 (9.6)	13.4	63.0
b_761_	761	756.2–766.7 (10.5)	14.7	71.9
b_802_	802	797.3–807.3 (10.1)	14.5	56.3
b_831_	831	826.3–835.8 (9.5)	13.1	55.3
b_861_	861	856.4–866.4 (10.1)	14.0	64.2
b_899_	899	891.3–907.7 (16.4)	22.9	63.6
b_953_	953	933.0–973.8 (40.8)	58.2	69.6

* FWHM = Full width at half maximum.

**Table 2:** Image digital numbers and ground measured reflectance of the white and black calibration panels in bands 1–12 and the respective regressions (empirical lines).

Band	DN Mini-MCA 12		Reflectance ASD Fieldspec		
	White Panel	Black Panel	White Panel	Black Panel	Regression
b_471_	1299.5	87.1	0.968	0.062	R = 0.000748 * DN − 0.003615
b_515_	1269.1	55.4	0.917	0.059	R = 0.000707 * DN + 0.019753
b_551_	1355.0	62.6	0.909	0.060	R = 0.000657 * DN + 0.018536
b_613_	1345.2	74.1	0.910	0.060	R = 0.000669 * DN + 0.009897
b_658_	1330.0	74.0	0.911	0.060	R = 0.000678 * DN + 0.010134
b_713_	1247.4	70.4	0.923	0.070	R = 0.000725 * DN + 0.018933
b_761_	933.6	64.9	0.936	0.080	R = 0.000985 * DN + 0.015794
b_802_	733.6	76.8	0.941	0.082	R = 0.001307 * DN − 0.018208
b_831_	722.7	71.6	0.943	0.083	R = 0.001321 * DN − 0.011693
b_861_	765.1	82.6	0.945	0.084	R = 0.001262 * DN − 0.020314
b_899_	656.6	75.7	0.947	0.084	R = 0.001485 * DN − 0.028555
b_953_	499.0	64.6	0.945	0.080	R = 0.001991 * DN − 0.048845

**Table 3:** *R*^2^, RMSE and MRE [%] for the relationships between the reflectance acquired by the 12 Mini-MCA bands and ground-based measurements at the 22 permanent observation sites.

	b_471_	b_515_	b_551_	b_613_	b_658_	b_713_	b_761_	b_802_	b_831_	b_861_	b_899_	b_953_
*R*^2^	0.16	0.10	0.04	0.19	0.40	0.11	0.88	0.91	0.90	0.89	0.88	0.84
RMSE	0.001	0.003	0.007	0.004	0.003	0.016	0.028	0.025	0.026	0.027	0.027	0.027
MRE%	51.2	104.4	58.0	33.0	82.6	22.7	4.0	3.6	4.3	3.8	4.4	4.6

**Table 4:** Yearly estimates of dry phytomass production and C export from four different Terrain/Soil type combinations (C1–C4) calculated from UAV imagery and ground based monitoring data.

			Monitoring 2014			UAV Mission (14-08-27)			
Case	Terrain position	Soil type (FAO)	Dry phytomass		factor	Dry phytomass		C export	
			4. harvest	per year		4. harvest	4. harvest	per year	
			[g·m^−2^]	[g·m^−2^]		[g·m^−2^]	[g·m^−2^]	[g·m^−2^]	CV [%]
C1	Steep slope	Calcaric Regosol	280	1409	5.03	288	124	624	21
C2	Flat hilltop	Albic Cutanic Luvisol	316	1452	4.60	363	156	718	17
C3	Midslope/hollow	Calcic Cutanic Luvisol/Endogleyic Colluvic Regosol	361	1556	4.32	376	161	697	14
C4	Midslope	Calcic Cutanic Luvisol-manipulated	307	1456	4.75	339	146	693	14
